# Orientation and disorientation in aviation

**DOI:** 10.1186/2046-7648-2-2

**Published:** 2013-01-03

**Authors:** John Richard Rollin Stott

**Affiliations:** 1 Aviation Medicine, King’s College London, Strand, London, England, WC2R 2LS, UK

**Keywords:** Spatial disorientation, Somatogravic, Oculogravic, Vestibular system, Aircraft accidents

## Abstract

On the ground, the essential requirement to remain orientated is a largely unconscious activity. In flight, orientation requires a conscious effort by the pilot particularly when the visual environment becomes degraded and a deceptive force environment becomes the frame of reference. Furthermore, an unusual force environment can determine the apparent location of objects within a limited visual scene, sometimes with disastrous consequences. This review outlines the sources of pilot disorientation that arise from the visual and force environment of flight and their interaction. It challenges the value of the traditional illusion-based approach to the subject both to aircrew and to surveys of disorientation. Also, it questions the emphasis on the shortcomings of vestibular function as the physiological basis for disorientation. While military accidents from all causes have shown a decline, there has been no corresponding reduction in accidents involving disorientation, 85% of which are the results of unrecognised disorientation. This finding has implications for the way in which pilots are taught about disorientation in the interest of enhanced flight safety. It argues for a greater use of conventional fixed base simulators to create disorientating scenarios rather than complex motion devices to create unusual sensations.

## Review

### Introduction

A sense of orientation is a fundamental requirement for all free-living creatures. The fact that it is a largely unconscious activity, like breathing, is some evidence of its physiological importance to everyday activities and even survival. The term orientation implies an awareness of self in relation to objects in one’s surroundings, either in the immediate environment or more remotely as a sense of geographic location. There is also an important sense of orientation within the body, namely proprioception. In flight, orientation refers more specifically to an awareness of the attitude and spatial position of the aircraft relative to the external frame of reference provided by the flat surface of the earth and the gravitational vertical. A pilot's sense of orientation cannot afford to be the unconscious activity that it is on the ground; he/she needs at all times to maintain an awareness of what the aircraft is doing.

The flight environment generates a number of hazards that relate to human physiology. For example, hypoxia at altitude and loss of consciousness during high G manoeuvres may have fatal consequences. The disorientating environment of flight may be less physiologically stressful, but the psychological stress of task saturation and the distraction of an in-flight emergency are important causes of accidents attributable to disorientation, a disproportionate number of which are fatal.

Why do pilots become disorientated? Many authors, in attempting to give a concise answer to this question, have fallen back on the statement that humans did not evolve to fly and that their sensory systems, in particular the vestibular system, are not adapted to the flight environment. The implication is that if only this system were a more perfect inertial navigation system, then flight without external visual reference would be less prone to orientation error. The problem with this explanation is that creatures that did evolve to fly share the same sensory systems, and in all probability the same limitations of those systems, as creatures that did not evolve to fly. Clear external vision is just as important to the flight of birds. Every falconer knows that a hooded bird will not fly. During the smogs that afflicted London before the Clean Air Act of 1956, it was observed that the London pigeons were grounded.

The limitation for unaided flight is not primarily a sensory problem but rather one of weight. Weighing about 10 kg, the swan is one of the heaviest creatures capable of independent flight, and it requires a long take-off run to gain sufficient speed to do so. The ostrich at 70–120 kg has, on an evolutionary timescale, long since given up the attempt, though its capacity to run at high speed, up to 70 km/h, may well be a legacy of the time when its somewhat less heavy ancestors required speed over the ground in order to become airborne.

So why do pilots become disorientated? In flight, there are two principal sources of orientation information: the visual environment and the force environment. There are circumstances in flight when a pilot may be inadequately informed by the external visual environment and deceived by the force environment. Furthermore, the force environment may influence how the pilot interprets the visual environment and where he perceives objects to be located within it.

Earthbound orientation is based on the assumption that the visual world is predominantly earth-stable and that the force experienced as gravity is constant in direction and intensity. Furthermore, there is an expectation of a relationship between the visual and the force world which requires, among many things, that the surface of lakes and seas are horizontal and that trees tend to grow vertically.

In flight, there are significant changes to the visual world, to the force world and to their interaction, which means that the earthbound assumptions no longer apply. Many of the problems associated with orientation in flight are the result of an unconscious misapplication by the pilot of the terrestrial rules of engagement that have been learned and depended upon from early infancy.

There is a well-worn dictum in aviation which states, ‘You cannot fly an aircraft by the seat of the pants’. This means that a pilot deprived of visual information cannot maintain his intended flight path by the feel of the aircraft alone. Early aviators did not appreciate this fact and were inclined to attribute any failure to maintain aircraft orientation when flying in cloud to a lack of ‘the right stuff’.

The decades preceding the dawn of powered flight had seen much progress in elucidating the function of the inner ear and, in particular, the motion-sensing capacity of the vestibular system [1]. It seemed natural therefore to suspect that disorientation in flight, which had become apparent from the loss of pilots and serviceable aircraft during World War I, was the result of deficiencies in the vestibular apparatus. In consequence, potential pilots were required to undergo tests of vestibular function and were excluded from training if the results fell outside certain limits [2]. It later became apparent that vestibular screening measures were not predictive of success in training or flying ability. At the same time, programmes were introduced to educate pilots about disorientation and to demonstrate vestibular-based illusions using the Barany chair. Elaborations of the rotating chair have continued to be used to the present day with the development of increasingly sophisticated and expensive devices in an attempt to replicate on the ground the motion and cockpit environment of an aircraft. Equally, vestibular function and its shortcomings have remained as a basis for the explanation of the numerous illusions of flight, perhaps to the neglect of other sensory mechanisms or considerations of the flight environment.

### The visual environment of flight

It is said that 80% of the information that a pilot needs in flight is acquired visually. Good foveal vision is required for object recognition; peripheral vision of a predominantly static visual environment is important for stability and orientation. However, the visual environment of flight can be deceptive. With increasing height above the ground, the visual sense of speed rapidly diminishes. Pilots who are used to flying at low level in fast jet aircraft, on transferring to a lower performance aircraft, may find themselves flying too low in an attempt to achieve their accustomed sense of speed over the ground.

When flying at low level, a pilot tends to judge height above the ground by the scale of objects in the field of view. This becomes more difficult when terrain features are largely absent as when flying over water, a desert or a snow-covered landscape. The pilot of a floatplane attempting to land on the glassy surface of a lake may be obliged to set up a descent rate of about 150 ft/min and wait until the aircraft touches down on the water rather than risk rounding out too soon or impacting the water at too high a descent rate. A similar approach may be required when landing on snow. Trees can be unreliable features against which to judge height. Stunted trees and bushes may lead to an overgenerous estimate of ground clearance.

Flight in mountainous terrain, particularly when snow-covered, may give rise to difficulties with height and distance estimation for lack of scale features. The horizon provided by the mountain tops is unlikely to represent the true horizon. There can also be problems in certain lighting conditions when the distant terrain masks the contour of more imminent high ground that is lying in the flight path of the aircraft. Similarly, a snow-covered ridge may become invisible against a background of uniform brightly lit cloud. White-out conditions have been a precipitating factor in a number of aircraft accidents, most notably when in 1979, an Air New Zealand DC10 crashed into the slopes of Mount Erebus on a sightseeing flight to Antarctica [3].

With increasing height above the ground, there is a loss of visual redundancy. Decisions about aircraft orientation are based on an increasingly restricted number of visual cues, any one of which may be misleading. For example, a cloudbank at lower level may obscure the true horizon and may lead to an increasing nose-down attitude. In hazy conditions, the pilot may align the wings with a gently sloping hillside. Flying at night, even in clear visual conditions, involves a profound loss of visual information compared with daytime flying. Stars and ground lights can be confused. Isolated stationary lights can appear to be in motion. Lines of light from street lights or along a waterfront may constitute convincing false horizons. It might be thought that a pilot who misaligned the wings with such a false horizon would be aware of the roll attitude error from a sensation of lateral tilt of the aircraft, but this is not so. An aircraft in an inadvertent banked attitude would enter a gentle turn and would continue to feel ‘wings level’—a phenomenon that is within the experience of all airline passengers.

Even in apparently clear visual conditions at ground level, with increasing altitude, the horizon may become hazy and indistinct, possibly to the extent that the pilot describes himself as flying in goldfish bowl conditions. In circumstance of deteriorating external vision, the pilot has to assess when the external visual cues are no longer sufficiently reliable and to make the decision to maintain aircraft attitude on instruments. Good instrument flying takes many hours to learn and constant practice to maintain. It involves the use of foveal vision to interpret the symbolic representation of the horizon in the attitude indicator, whereas visual orientation is predominantly the role of peripheral vision. With no external visual cues, the pilot is only aware from peripheral vision of the apparently stable interior of the aircraft cockpit. This represents a reversal of the respective roles of central and peripheral vision.

### The force environment of flight

Newton's third law states that for every force, there is an equal and opposite reaction. This means that all forces are *interactions* between different bodies; there is no such thing as a unidirectional force or a force that acts on only one body. The downward force that we experience as the force of gravity only exists as a consequence of the upward force exerted by the floor. This upward force acting on the body gives the perception of weight and defines what we sense as the vertical. During running and jumping, when both feet are off the ground, the body is transiently weightless. An aircraft in flight only acquires weight by virtue of the aerodynamic force acting on the wings. This force, known as lift, is related to wing area and is proportional to the square of the speed through the air and, up to a certain limit, the angle of attack—the angle that the wing makes relative to the airflow. Both airspeed and angle of attack are under the control of the pilot through the throttle and the control column. As a consequence, it is the pilot that determines the weight of the aircraft and the sense of gravity experienced by the occupants not only in terms of its intensity, but also, by changes in aircraft attitude, its direction. The effect of aerodynamic force is to impose on the aircraft its own force vertical which is not necessarily aligned with the earth vertical. The mistaken assumption by a pilot that what is felt as gravity is truly vertical underlies a significant number of disorientation incidents and accidents.

The effect of the gravitational attraction of the earth imposes the same physical constraint on the pilot of an aircraft as on earthbound individuals; the component of force acting in the earth vertical direction must average over time to the static weight of the object, human body or aircraft. Any period for which this component of force is decreased must be paid for by a corresponding period of increased vertical force. The difference between airborne and earthbound man is in the longer time period for which this rule can be infringed. Given sufficient height above ground, an aerobatic pilot can enjoy a gravitational freedom that is denied mere earthbound mortals.

**Figure 1:**
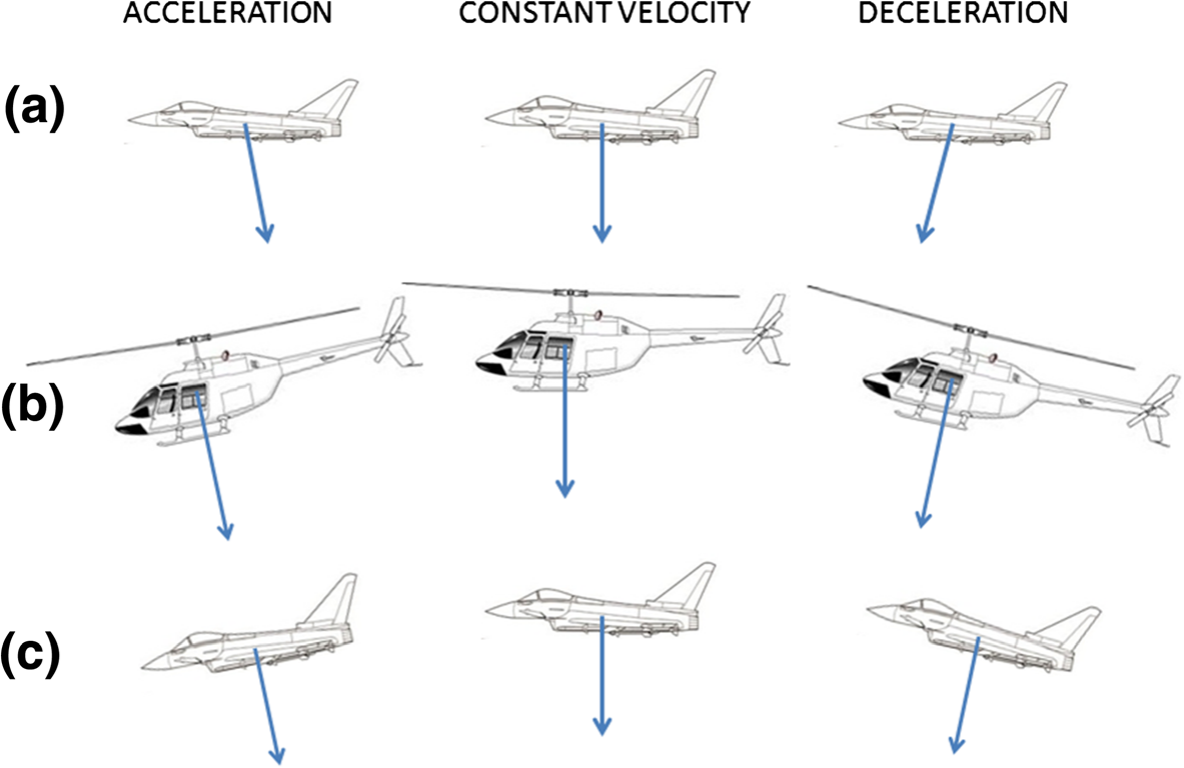
**The somatogravic effect—the non-visual sensations of aircraft attitude.** The *arrows* indicate the magnitude and direction of the resultant force vector generated by powered fixed-wing aircraft and rotary-wing aircraft during acceleration, constant velocity flight and deceleration. In the fixed-wing aircraft (**a**), the resultant force is derived from the addition of two forces—the lift on the wings and the force associated with acceleration or deceleration in the line of flight. To the aircraft occupants, the resultant force establishes a sense of the vertical that is no longer aligned with the true vertical. In consequence, a powered aircraft when accelerating tends to feel more pitched up and when decelerating, more nose down than it actually is. In the helicopter (**b**), the lift of the main rotor is the only source of force for both lift and forward acceleration or deceleration. Forward acceleration can only be achieved by putting the helicopter into a nose-down attitude. However, the force from the rotor remains predominantly vertical with respect to the aircraft. In consequence, a helicopter feels to be in a level attitude whether it is accelerating, at constant velocity or decelerating. A fixed-wing aircraft (**c**) can also feel to be in a level attitude as a consequence of a possibly inadvertent nose-down or nose-up attitude when unaccompanied by any change in engine power setting since the change of attitude alone results in acceleration or deceleration in the line of flight. This phenomenon is similarly experienced in an unpowered glider.

A further force on a fixed-wing aircraft that acts in the long axis of the aircraft approximately at right angles to the lift force comes from the thrust of the engines and the retarding effect of the airbrakes. Though this force contributes to problems with spatial orientation during changes in airspeed, its intensity in most aircraft is substantially less than that of gravity (Figure 1a). In a helicopter, there is no similar longitudinal force. The force that both lifts the aircraft off the ground and drives it forward (or backward or sideways) is generated by the main rotor, and this force is always vertically upward relative to the fuselage of the helicopter, whatever the aircraft attitude may be with respect to the surface of the earth (Figure 1b).

These examples form part of a group of disorientating problems known as somatogravic effects or illusions. Specifically, the term ‘somatogravic’ is applied to situations in which the combined forces acting on the aircraft are perceived as gravity and are erroneously assumed to indicate to the pilot the true earth vertical.

The somatogravic effect is important for two reasons. First, it is involved in almost every aircraft manoeuvre and provides an explanation of why, in the absence of external vision, a pilot can readily be deceived by what he feels the attitude of his aircraft to be. Second, it is an underlying factor in many aircraft accidents. This effect is responsible for situations in which the aircraft continues to feel level despite being either in a banked attitude or pitched up or pitched down, and also situations in which the aircraft is felt to be pitched up or down to a greater extent than it actually is.

**Figure 2:**
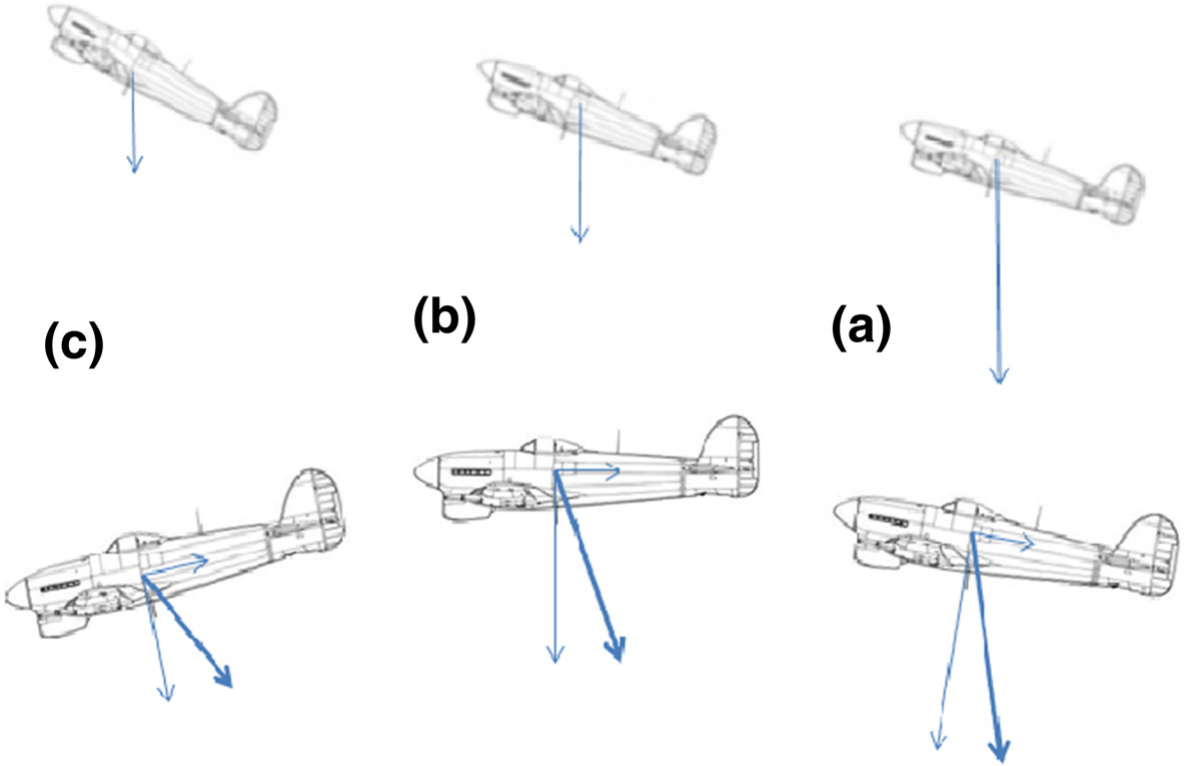
**The dark night take-off accident.** An aircraft accelerating in the climb feels to the pilot to be climbing more steeply than intended (**a**). To correct this impression, the pilot pushes forward on the control column with the result that the aircraft may no longer be climbing (**b**) and with continued inappropriate control, may begin to descend (**c**). In the force vector diagram associated with each of the lower aircraft pictures, the constant thrust of the engine is represented by the backward inertial force that it generates, while the lift on the wings, which decreases as a consequence of the aircraft trajectory, is represented by an inertial force vector acting vertically downward with respect to the aircraft. The resultant vector representing the sum of these two forces is shown in bold. In the absence of clear external vision, as might occur on a dark night or a take-off into cloud, this resultant force can be perceived as the gravitational vertical with implications for the pilot's perception of the pitch attitude of the aircraft. Each image in the upper series has been rotated to align the resultant force vector with the true vertical so as to represent what the pilot might think is happening to the aircraft. It can be seen that the pilot's inappropriate control action may increase rather than decrease the perception of pitch-up of the aircraft.

A type of aircraft accident that was recognised during World War II became known as the dark night take-off accident [4] in which, after take-off on a night with few external visual cues, the aircraft was flown into the ground at a shallow angle some distance beyond the end of the runway. The forward acceleration of a fixed-wing aircraft during and after take-off combines with the lift on the wings to generate a net force that is no longer aligned with the vertical and leaves the pilot with a sensation of a steeper climb angle than is actually the case (Figure 2). The response of the pilot may be to lower the nose of the aircraft. However, this action is unlikely to reduce the sensation of excessive pitch-up as it allows the aircraft acceleration to increase and thus intensifies the illusory pitch-up at the expense of actual pitch attitude. Since its recognition, there have been many examples of this type of accident, either at night or following take-off into cloud. It can also be a problem when, after a missed approach to land, often in bad weather, the pilot is obliged to go around. This manoeuvre involves an immediate increase in thrust from the engines which may leave the pilot with the reassuring sensation that the aircraft is climbing when it is not. There is a corresponding effect from deceleration of the aircraft from the use of airbrakes which leads to an illusory sensation of pitch-down. An inappropriate nose-up response by the pilot is possibly less dangerous but may lead to a significant loss of airspeed and a possible stall.

The somatogravic effect thus described occurs in association with deliberate manoeuvres initiated by the pilot and is therefore amenable to training programmes to guard against inappropriate control actions. There is, however, what may be termed an inadvertent somatogravic effect in which a pilot, distracted while flying straight and level, inadvertently allows the nose of the aircraft to drop (Figure 1c). In this attitude, the aircraft will accelerate and the consequent sensation of upward pitch will negate the actual pitch-down of the aircraft and leave the pilot with the sensation that the aircraft remains straight and level.

The inadvertent somatogravic effect can occur in all aircraft but is a specific problem in helicopters. A helicopter has no source of force in the longitudinal direction of the fuselage. In order to accelerate in the line of flight, a helicopter has to pitch down so that a component of the lift force generated by the rotor acts in a forward direction. The illusory sense of pitch-up that accompanies forward acceleration almost exactly compensates the actual pitch-down required to achieve that acceleration, and in consequence, the aircraft continues to feel level. Likewise, in order to reduce airspeed, the helicopter pilot has to put the aircraft into a nose-up attitude so that a component of lift acts in a backward direction. The pitch-down effect of deceleration negates the actual pitch-up. An alternative way to explain this effect is to recognise that, neglecting the tail rotor, the only source of aerodynamic force is the force exerted by the main rotor. Its predominantly upward pull creates a sense of gravity within the aircraft that remains vertically downward with respect to the aircraft, whatever its attitude, relative to the true vertical. It is true that to initiate a change of attitude in pitch or roll, the pilot has aerodynamically to tilt the rotor with respect to the fuselage, but this is only transient. The fuselage soon follows the alignment of the rotor disc. Also, it is important to recognise that the somatogravic effect is not evidence of shortcomings in the gravity sensors within the inner ear; accelerometers would give the same indication.

A sensation that is frequently described by instrument-rated pilots may follow a period of manoeuvring in cloud, when the aircraft is felt to be flying one wing low despite straight and level being indicated on the attitude instrument. From its tendency to make the pilot lean towards the erroneously perceived vertical, this effect is known as ‘the leans’. As the aircraft is flying straight and level at constant airspeed, no additional force other than that to oppose gravity is acting on the aircraft. In consequence, the leans would appear to require a physiological or perceptual rather than an aerodynamic explanation. The leans disappear the moment there is a clear external visual scene but are not readily dispelled while ever an awareness of aircraft attitude is entirely derived from the aircraft instruments. Once pilots become aware of the discrepancy between their sensations of the roll attitude of the aircraft and the indication of the artificial horizon, their training leads them to obey the instruments and to disregard what can sometimes be a very distracting sensation. In consequence, they remain aware of the orientation of the aircraft, and there is seldom any risk to flight safety.

### The vestibular system

The vestibular labyrinth of the inner ear is an important sensor of the force environment. Its two functional components sense linear forces, in particular the force of gravity, through the otolith organs and angular forces through the semicircular canals. The sensory information generated by the vestibular system contributes to the maintenance of postural stability and balance and, more exclusively, to the stabilisation of the retinal image through the vestibulo-ocular reflex. Individuals who lack vestibular function find that with any movement of the head, they perceive a moving and therefore somewhat blurred image of the stable visual world, which is termed oscillopsia [5]. Although popularly known as the balance organ, it shares this function with the visual system and with kinaesthetic sensors within the muscles, tendons and skin. On account of its detrimental effect on vision, a pilot with a total vestibular paresis would be grounded. However, in other respects, compensatory mechanisms would minimise the effect on balance, both on the ground and in the air. A pilot who developed total vestibular paresis following treatment with an aminoglycoside antibiotic successfully flew RAF transport aircraft for 10 years before the diagnosis was made. Similarly, a laboratory study of the sensation of tilt produced by the somatogravic effect showed no difference between normal and labyrinthine defective individuals [6].

The physiologists, Mach, Breuer and Crum Brown, working independently in the 1870s established that the stimulus to the semicircular canals was the movement of fluid within them and that the neural transduction process arose from the detection of movement by hair cells within the vestibular labyrinth, similar to those found in the cochlea. Hair cells have a directional sensitivity. Deflection of the hairs determines the rate of depolarisation of the cell which is dependent on mechanically gated ion channels at the tips of each hair [7].

The semicircular canals communicate at each end with the utricular cavity and thus each canal forms a complete ring of endolymphatic fluid interrupted only by the cupula. This gelatinous membrane with its embedded hair cells lies across the ampulla, a dilated portion of the canal near its junction with the utricle, and acts as a sensor of fluid movement. The function of the semicircular canal depends on three elements, the inertia of the endolymph, the elasticity of the cupula and the viscous drag on fluid movement generated by the wall of the canal. This resistance to the flow of endolymph through a canal that is only 0.4 mm in diameter exerts the dominant effect on the system. The behaviour of the semicircular canal system is well described by a second-order dynamic model, the torsion pendulum model [8], which has received experimental verification in fish, amphibians, birds and primates [9]. The torsional acceleration imposed on the canal determines the rate of fluid movement along the canal. The compliance of the cupula allows its displacement to be an indicator of the amount of fluid that has accumulated in one half of the ampullary chamber and drained from the other half. The canal system thus becomes a sensor of the accumulation of angular acceleration, which is, by definition, angular velocity.

**Figure 3:**
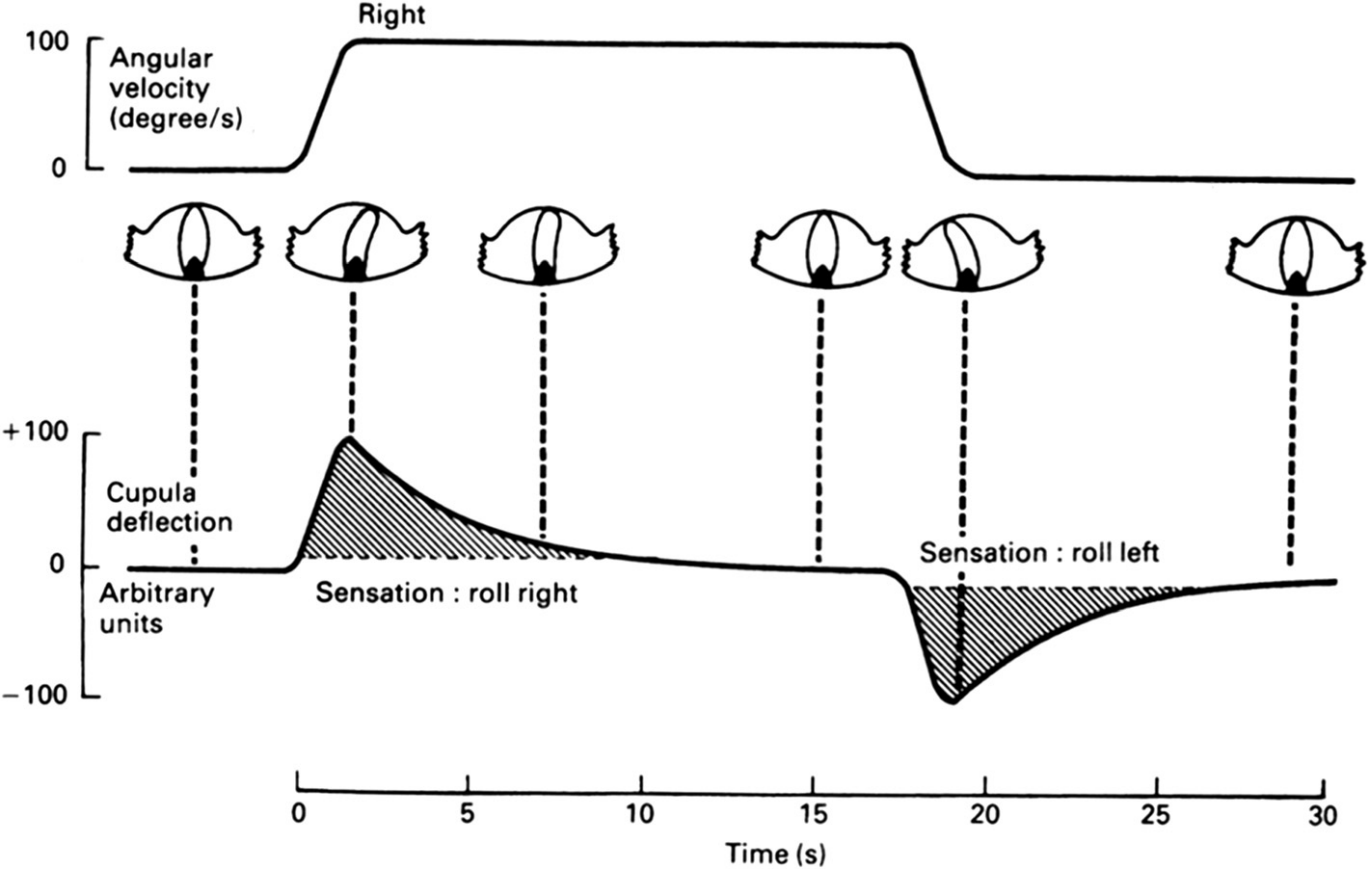
**Response of the semicircular canal to sustained angular velocity.** The increasing angular velocity as a consequence of an angular acceleration over a 2-s period is accurately sensed. If a constant angular velocity is maintained, the cupula gradually returns to its central position, and there is an exponential decay in the sense of rotation. If then a deceleration is initiated, it is sensed as an exponentially decaying sense of rotation in the opposite direction.

For the short-duration angular accelerations and decelerations that occur during normal head rotations, this system sends an accurately coded neural representation of angular velocity to the brain. However, if angular acceleration is prolonged, the resulting angular velocity is progressively underestimated on account of the increasing elastic resistance of the cupula to fluid movement. Cupular deflection is only maintained for as long as angular acceleration continues. If, following acceleration, rotation is held at constant velocity, the elasticity of the cupula slowly restores it to its central position, retarded by the return flow of endolymph around the canal, and in consequence, there is an exponentially decaying signal of rotational velocity. When rotation ceases after a period at constant velocity, the cupula having returned to its central position, the angular deceleration produces a cupular deflection and a signal of rotation in the opposite direction (Figure 3). The illusory sensation of rotation is known as the somatogyral effect and its visual counterpart, the apparent rotation of the visual scene, as the oculogyral effect.

A prolonged period of rotation is a feature of some aircraft manoeuvres, notably spinning and aerobatics. Such manoeuvres require a clear visual appreciation of the motion of the aircraft, preferably from a clear external view.

In the laboratory, using a high-fidelity turntable, it is possible to measure a threshold for the perception of rotation. For angular acceleration lasting less than 10 s, rotation will not be perceived until the product of acceleration and the time for which it is applied exceeds 2.5° .s^−1^. Known as Mulder's law, this defines a perception threshold in terms of rotational velocity and reflects the function of the canal as an integrating accelerometer. In the flight environment, the threshold of detection of rotation is likely to be much greater, particularly if a pilot is distracted. Also, a rotation in pitch or roll is less likely to be detected for lack of any confirmatory sensation of increasing tilt.

Lying within the utricular and saccular cavities are the maculae; in the utricles, they lie roughly in the horizontal plane; in the saccules, in the sagittal plane. Each macula consists of a carpet of hair cells overlain by an otolithic membrane. Within this membrane are otoconia, crystals of calcium carbonate between 0.5 and 30 μm in diameter. The otoconia cause the membrane to be denser than the surrounding endolymph and consequently subject to movement over the macula from the effect of gravity and the inertial forces associated with locomotion. There is a progressive change in the alignment of the axis of maximal hair cell sensitivity across the surface of the macula. As a result, a force applied in any direction within the plane of the macula will preferentially stimulate certain groups of hair cells. This anatomical arrangement of the utricular and saccular maculae and the alignment of the hair cells within them enable the otoliths to sense the amplitude and direction of any external force. In effect, they are multidirectional accelerometers.

### The oculogravic effect

In everyday life, the ability to locate an object in the field of view can generally be derived from its visual context. Such cues as size and position relative to other objects, the effect of parallax that results from head movement and, for nearer objects, the effect of stereopsis produce multiple pieces of evidence on which to derive an accurate perception. This perception is confirmed by the sense of the vertical generated by the effect of gravity on the body. The frame of reference may appear to be predominantly visual, but there is an associated concordant gravitational frame of reference. In an aircraft, particularly when flying on a moonless night, circumstances can arise in which the object of interest, such as a distant runway or the lights of an oil platform, lacks any useful visual frame of reference. In these circumstances, a pilot may unconsciously fall back on the force frame of reference provided by the aircraft. It has already been discussed how a force frame of reference is generated by the aerodynamics of the aircraft and how a pilot can assume that it represents a true vertical. If the force frame of reference is deceptive, so too may be the apparent location of the object or of the aircraft relative to it.

**Figure 4:**
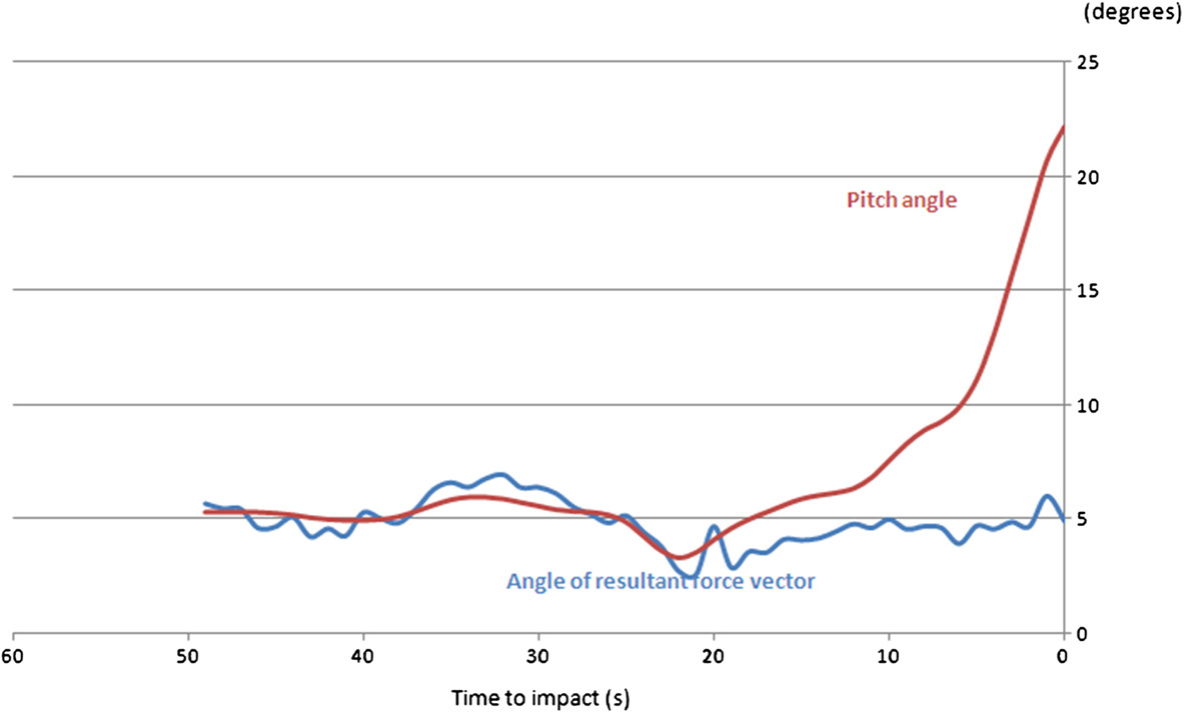
**The dissociation between tilt and pitch attitude change in a helicopter.** While the aircraft developed an increasing pitch-up attitude during the final 10 s of flight, there was no corresponding change in the angle of the combined Gx and Gz force vectors as measured by on-board accelerometers. The aircrew would have had no sensation of backward tilt to alert them to the increasing pitch-up attitude of the aircraft.

**Figure 5:**
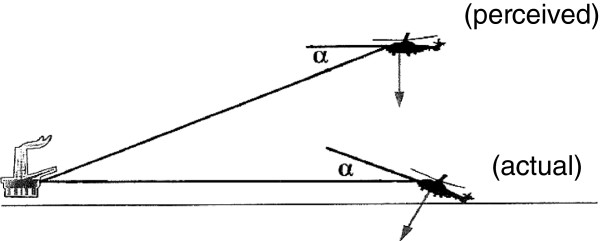
**The oculogravic effect.** In the absence of any visible horizon and probably of any depth perception associated with the lights of the oil platform, the pilots were unaware of the increasing pitch-up attitude of the aircraft, which had reached 22° at the point of impact with the sea (*angle α*). They had unconsciously used the sensation of level flight generated by the helicopter dynamics as the frame of reference against which to judge their position relative to the platform. In consequence, they had perceived the platform to be below them.

This situation is exemplified by an accident to a helicopter on the approach at night to a North Sea oil platform [10]. Conditions were calm, but the approach was complicated by fog in the vicinity of the platform. The moon was below the horizon, and stars were obscured by cloud. There would have been no visible horizon. When about 400 m from the platform, the aircraft was at a height of 420 ft and began to descend and turn towards the platform. On the final approach, both pilots were looking out in an attempt to identify the green perimeter lights of the helideck at an elevation of 166 ft above the sea when the aircraft unexpectedly impacted the sea with a nose-up attitude of 22° some 300 m short of the platform. The accident involved no loss of life, in large measure, due to the fact that an increasing but unperceived nose-up attitude had reduced the forward airspeed to about 20 kt at the moment of impact. In the few seconds before impact, the pilots reported that, far from thinking themselves to be below the level of the helideck, they had a visual impression that they were high above the platform and about to overshoot it. This marked discrepancy between the perception and the true situation had come about from the sense of level flight derived from the force environment of the aircraft having become the frame of reference against which the pilots judged the visual environment. The data shown in Figure 4 are derived from the flight data record of the pitch attitude of the aircraft and from the combined accelerometer recordings in the fore–aft and aircraft vertical directions. They illustrate how the change in pitch attitude had no effect on the linear force environment of the aircraft and thus led to the pilots' increasing misperception of the true gravitational vertical (Figure 5).

Another helicopter accident had occurred a few years previously in similar night time conditions when approaching to land on a gas platform in Morecambe Bay [11]. During the approach, the handling pilot inadvertently flew the aircraft into an attitude of 38° of roll and 38° nose down. Using the flight data records, it could be shown that despite this slowly developing unusual attitude, the lights of the platform on which they were to land would have appeared in the same place on the windscreen—a seemingly appropriate approach strategy. Expert opinion from experienced helicopter pilots asserted that the unusual attitude would have *felt* very alarming. In truth, it would only have *appeared* very alarming had there been anything else to see outside the aircraft other than the two-dimensional pattern of lights of the platform against a uniformly black background. Again, it could be shown from the flight data records that the dynamics of the aircraft would have ensured that the aircraft continued to feel level and that the rotations in pitch and roll would have been too gradual to be sensed.

In both these accidents, there was a failure to monitor aircraft instruments together with a lack of appreciation that, in the prevailing visual conditions, the isolated target that the pilots could see might not be where it appeared to be. In the disorientation literature, this visual counterpart of the somatogravic effect is not always well described and its particular relevance to helicopter operations not fully appreciated.

A further manifestation of an oculogravic effect is described by helicopter pilots attempting a night landing at a remote site, guided by an illuminated T laid out on the ground. As the aircraft manoeuvres in its approach, if there are no other visual cues in the immediate vicinity, the T is seen to wander in the pilot's gaze. The pilot's perception is of a moving visual target with reference to a fixed force environment rather than the reverse.

The apparent slope of a true horizon during a banked turn is another example of an oculogravic effect. The sense of gravity that remains vertical with respect to the aircraft acts as the frame of reference that perceptually tilts the horizon.

Airline passengers who find themselves seated in an aisle seat at the rear of the aircraft might be interested to make the observation that, while the aircraft is accelerating along the runway during take-off and well before the pilot raises the nose of the aircraft to take to the air, the horizontal floor of the aircraft cabin appears to slope upward—an oculogravic effect. In addition, if anybody were permitted to walk forward along the aisle during the take-off run, they would feel as though they were walking uphill—the corresponding somatogravic effect.

### The relationship between disorientation and illusions of flight

Disorientation presents to a pilot in just one of two ways: either there is a sense of confusion about the attitude of the aircraft on account of deteriorating visual information and an awareness of conflicting sensations, or everything feels as expected until there is a sudden realisation that the aircraft is not in the attitude or position that it was intended to be.

When a pilot makes an error in the attitude or spatial position of the aircraft, the pilot is said to be disorientated and to have suffered an illusion. The term ‘illusion’ is widespread throughout the literature on spatial disorientation, and the nature of the underlying illusion has tended to form the basis of classification of disorientating in-flight events. Its definition within psychology as ‘a misinterpretation of an experience of sensory perception, especially a visual one, where the stimuli are objectively present and the mistaken perception is due to physical rather than psychological causes’ is entirely appropriate to the subject of disorientation. However, a pilot can be aware of an illusion without necessarily being disorientated. The two terms are by no means synonymous. Illusions in flight are universal, whether or not a pilot notices them; the loss of awareness of speed with increasing altitude, the nose-up sensation during forward acceleration, the feeling of wings level during a banked turn are present every time an aircraft flies. By contrast, disorientation incidents are relatively rare. To be aware of an illusion requires a simultaneous appreciation of the perceptual error and the reality. It could therefore be argued that once a disorientated pilot becomes aware of the illusion, he/she is no longer disorientated.

A further problem associated with an illusion-based approach to disorientation is that it gives no indication of the likelihood that a given illusion will lead to a disorientation incident or accident. The illusion that a banked aircraft continues to feel straight and level is probably the most frequent predisposing condition for disorientation episodes, yet this illusion hardly merits inclusion in any list of illusions of flight; it is just too commonplace. If a distracted pilot inadvertently allows the aircraft to develop a slowly increasing angle of bank while still maintaining the same degree of lift required for level flight, the vertical component of the lift force will become increasingly inadequate, and the aircraft will begin to lose height. With further increase in bank angle, the aircraft, though still feeling to the pilot to be in level flight, will develop a nose-down attitude and enter a spiral dive with an accelerating loss of height. This scenario is known as the graveyard spiral. Surveys of pilot experience of spatial disorientation have tended to concentrate on the incidence of known in-flight illusions [12]. There are several difficulties with this approach. One problem was highlighted by an unpublished survey of 100 pilots who were asked to report their most recent experience of disorientation and its impact on flight safety. In four of the five reported incidents in which flight safety was considered at risk, the pilots were unable to say what illusion they had suffered. Another illusion-based survey concluded that ‘pilots who had received in-flight [spatial disorientation] SD training reported more episodes of SD than those who had not participated in this training’ [13]. It is hardly likely that in-flight training had increased the incidence of disorientation. It is more likely that what is described as SD in this context had been confused with an awareness of illusions.

In an ongoing survey of disorientation, military pilots were asked to give an account in their own words of events in flight when they had become confused about the attitude or spatial position of the aircraft or had suddenly become aware that the aircraft was not in the attitude they expected it to be. It was considered that a survey of this type would give a truer picture of the functional significance of the disorientating aspects of flying. A significant finding was that in over 300 incident reports, the word ‘illusion’ was mentioned only once and then only in the pilot's retrospective assessment of the incident. It must be concluded that a disorientated pilot does not *experience* an illusion.

### Disorientation accidents and incidents

A common theme in spatial disorientation incidents, and, by extension, accidents, is a preoccupation with one aspect of the flying task to the exclusion of accurately flying the aircraft, sometimes necessitated by an in-flight emergency or a period of excessively high workload. Many pilots have commented on how quickly a flight trajectory can go from safe to unsafe when attention is diverted away from the flying task. This is particularly true when the aircraft is manoeuvring at low level.

Analysis of accidents often reveals multiple causal factors leading up to the final event. An assessment of the role played by disorientation in any accident may have to rely on circumstantial evidence, such as knowledge of weather conditions at the time and the manoeuvre being attempted, in order to arrive at a conclusion. The conclusion is always open to investigator bias. For these reasons, accident surveys, almost all of them dealing with military accidents, vary quite widely in the reported percentage of accidents attributable to spatial disorientation.

A recent survey of UK military accidents [14] covering the two decades, 1983–1992 and 1993–2002, showed a reduction in the rate of all accidents, particularly in rotary-wing aircraft, between the two time periods but little change in the rate of spatial disorientation-related accidents (Table 1). Several factors were identified that increased the relative risk of a disorientation-related accident. Risk was increased by a factor of 2 when night flying and by a factor of about 3 if flying in cloud or in degraded visual conditions. Failure of communication within the cockpit led to an almost fourfold increase in risk. Of particular significance in this survey were the findings that 50% of disorientation-related accidents involved distraction and that, at the point at which the accident became inevitable, disorientation remained unrecognised in 85% of accidents.

**Table 1:** Spatial disorientation in military aircraft accidents from 1983–2002

**Accident rates per 100,000 flying hours**				
**Aircraft**	**Accident**	**1983–1992**	**1993–2002**	**Reference**
Fast jet	All accidents	7.0	5.8	
	Disorientation- related accidents	1.7 (24.2%)	1.6 (28.2%)	
Rotary wing	All accidents	4.1	2.4	[14] Bushby
	Disorientation-related accidents	1.0 (24.3%)	1.0 (42.2%)	

Military accident rates per 100,000 flying hours for all accidents and for those in which spatial disorientation was a factor. Comparison of the two 10-year periods, 1983–1992 and 1993–2002, shows a fall in overall accident rate, more evident for rotary-wing than for fixed-wing aircraft, but there was no decrease in the incidence of disorientation-related accidents. For comparison, the accident rate for scheduled commercial aircraft with a maximum take-off weight (MTOW) greater than 2,500 kg is 0.4 per 100,000 departures [15].

In the survey of disorientation incidents referred to in the previous section, it was found that, of those incidents rated by the reporting pilot as significant or severe in relation to flight safety, 75% involved unrecognised disorientation. This close correspondence with the 85% of disorientation accidents that involved unrecognised disorientation is some evidence that the incident survey is collecting reports of potential accidents, the circumstances of which could be of value in the disorientation training of aircrew.

### Aircrew training in the prevention of spatial disorientation

It is of obvious importance to warn aircrew of the potential dangers of disorientation and that the aircraft attitude is consciously to be monitored rather than taken for granted. Training for disorientation has traditionally been part of an aeromedical training programme. While this is appropriate in view of the physiological limitations that are involved, the aerodynamic behaviour of the aircraft is also a major contributor to potential spatial disorientation. Furthermore, the remedy for the condition rests with the pilot and his training to fly the aircraft so as to minimise the risk of disorientation. Medical advice on the avoidance of disorientation is inevitably general in nature. The more specific aspects of disorientation training are the responsibility of the flight instructors.

In addition to lectures and video presentations, aircrew are given the opportunity to experience spatial disorientation in a dedicated simulator. These devices have evolved from relatively simple rotating chairs to enclosed rotating cabins with visual presentations of an aircraft instrument panel and an external visual scene. The aim of the earlier devices was to demonstrate the fallibility of vestibular sensors of motion, particularly to rotational stimuli. Rotation about a vertical (yaw) axis has remained a feature of later disorientation training devices, and subjects are encouraged to experience the disturbing Coriolis sensations that accompany head movements in this environment. However, such sensations induced on the ground may far exceed any equivalent sensations in flight where any yaw axis rotation is usually of low intensity. One solution to a possible negative transfer of ground-based training to the flight environment has been the adoption by some air forces of dedicated flights designed to demonstrate manoeuvres that can deceive the pilot.

The problem of unrecognised disorientation, the situation in which everything feels normal despite a worsening deviation from the intended flight path, is not well addressed by ground-based disorientation simulators. The confusing sensations that they demonstrate, if experienced in flight, act as the trigger for the trained pilot to rely on the aircraft instruments to determine the true situation. However, the great majority of disorientation-related accidents occur without the alerting benefit of confusing sensations. For these, it is necessary to train pilots in the circumstances, such as go-around, take-off into cloud, low-level abort manoeuvres, in which the risk of unrecognised disorientation is high. An equally important aspect of disorientation training is prioritisation of tasks and the apportionment of time devoted to them in relation to the demands of maintaining an accurate flight path. These considerations are well suited to the use of modern training simulators in which the motion environment is far less important than the ability to create a large range of scenarios that tax the pilot's airmanship with an increasing workload and create the potential for unrecognised disorientation. This use of conventional simulators is being introduced for disorientation training in both fixed- and rotary-wing aircraft at multiple stages throughout a pilot's career. Whether this development will have a beneficial impact on the accident statistics, only time will tell.

Though not affecting the incidence of pilot disorientation, there are technological solutions available to reduce the worst consequences of it in accidents in which a serviceable aircraft is flown into the ground. Ground proximity warning systems have long been a standard feature of commercial and military aircraft. Incident reports have confirmed their value in alerting the pilot to a dangerous situation, but they require the pilot to respond appropriately and cannot anticipate an unrecoverable aircraft attitude or menacing terrain features. The Eurofighter Typhoon aircraft is fitted with an automatic recovery system. The publicity material states, ‘In the unlikely event of pilot disorientation, Eurofighter Typhoon's [flight control system] FCS allows for rapid and automatic recovery by the simple press of a button.’ Such a system requires a pilot to recognise that he/she is disorientated before pressing the button. It is therefore unlikely to be of much value in the 85% of disorientation accidents attributable to unrecognised disorientation. The ultimate solution may be provided by automated ground collision avoidance systems. Their widespread use is made possible by computer software incorporating detailed terrain maps and the availability of accurate aircraft position information derived from global positioning systems.

## Conclusions

Much of the basic physiological science of relevance to disorientation in aircraft has been elucidated many decades ago, some even before the advent of powered flight. The practical problem remains as to how the subject should be taught and demonstrated to each successive generation of pilots to forewarn them and maintain their awareness of the potential dangers of disorientation in flight.

## Competing interests

The author declares that he has no competing interests.

## Authors’ information

JRRS is an honorary senior lecturer in Aviation Medicine at King’s College, London and formerly a senior medical officer at the RAF Institute of Aviation Medicine and its successor organisations, DERA and QinetiQ plc.
